# Exercise ventilatory efficiency in elite athletes assessed for the Paris 2024 Olympic Games: The effect of sex and sport categories

**DOI:** 10.14814/phy2.70261

**Published:** 2025-04-02

**Authors:** Maria Rosaria Squeo, Ilaria Menichini, Matteo Morviducci, Alessandro Spinelli, Giuseppe Di Gioia, Viviana Maestrini, J. Alberto Neder, Paolo Palange

**Affiliations:** ^1^ National Italian Olympic Committee (CONI) Institute of Sport and Science Medicine Rome Italy; ^2^ Department of Public Health and Infectious Disease, Respiratory and Critical Care Division Sapienza University of Rome Rome Italy; ^3^ Department of Cardiovascular Sciences Campus Bio‐Medico University Rome Italy; ^4^ Department of Clinical, Internal, Anesthesiological and Cardiovascular Sciences Sapienza University of Rome Rome Italy; ^5^ Respiratory Investigation Unit, Division of Respirology, Department of Medicine Queen's University and Kingston Health Science Center Kingston Canada

**Keywords:** CPET, elite athletes, exercise, ventilatory efficiency

## Abstract

Ventilatory efficiency during cardiopulmonary exercise testing (CPET) is obtained by relating minute ventilation (V'_E_) to CO_2_ output (V'CO_2_). Limited information is available regarding exercise ventilatory efficiency in young elite athletes. We assessed ventilatory efficiency in elite athletes; evaluating the influence of sex and/or ESC sport categories; evaluating the agreement between the V'_E_/V'CO_2_ slope and nadir in measuring ventilatory efficiency; evaluating differences between subgroups of athletes stratified by ventilatory efficiency. A cohort of 443 elite athletes prospectively underwent CPET. The slope (s_1_) and the intercept of the linear region of the V'_E_/V'CO_2_ relationship, the V'_E_/V'CO_2_ value at the lactate threshold and the V'_E_/V'CO_2_ nadir were used to assess ventilatory efficiency. Male athletes and endurance athletes, both males and females, had higher exercise ventilatory efficiency (*p* < 0.001). A strong positive correlation was observed between V'_E_/V'CO_2_ s_1_ and nadir (*p* < 0.001). Of note, both high (V'_E_/V'CO_2_ s_1_ < 24) and very high (V'_E_/V'CO_2_ s_1_ < 22) levels of ventilatory efficiency were associated with greater exercise tolerance (i.e., peak oxygen uptake, maximal power; *p* < 0.001). The results of our study emphasize the need to include the measurement of ventilatory efficiency in the evaluation of elite athletes, potentially refining their training strategies.

## Graphical abstract

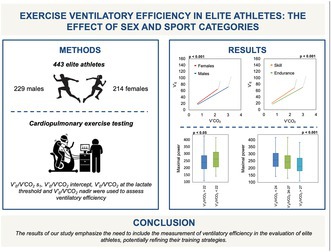

This graphical abstract illustrates the role of ventilatory efficiency (V'_E_/V'CO_2_) in elite athletes, analyzed by sex and sport category.

## 1 INTRODUCTION

Cardiopulmonary Exercise Testing (CPET) is the gold standard for the assessment of aerobic fitness, allowing the evaluation of the integrated cardiovascular, respiratory, and metabolic response to incremental exercise (American Physical Therapy Association, 2001; ERS Task Force et al., 2007). Currently, the most widely used parameter in sports‐related research regarding athletes' fitness is peak oxygen uptake (V'O_2_ peak) (Petek et al., 2022). On the contrary, there is limited information available concerning the impact of exercise ventilatory response and ventilatory efficiency on exercise performance in elite athletes (Petek et al., 2021, 2023; Salazar‐Martínez et al., 2018). This is a subject of particular importance for this population where even modest uncomfortable exertional sensations—such as breathlessness caused by excessive ventilation (Neder et al., 2022)—may negatively impact maximal performance.

In this context, it is well known that the rate of the increase in minute ventilation (V'_E_) over carbon dioxide output (V'CO_2_) reflects the subject's “ventilatory efficiency” (Di Paolo et al., 2019; Neder et al., 2017; Ramos et al., 2013). Specifically, each exercise level of minute ventilation (V'_E_) corresponding to a particular V'CO_2_ is inversely related to the “set point” at which arterial CO_2_ partial pressure (P_a_CO_2_) is controlled and influenced by the physiological dead space fraction of tidal volume (V_D_/V_T_) according to the following equation (Ramos et al., 2013):

$$\frac{V^{\prime }E}{V\prime {\mathrm{CO}}_{2}}=\frac{1}{{\text{PaCO}}_{2}\left(1-\left(\frac{V_{D}}{V_{T}}\right)\right)}$$

At very low exercise intensity, a slight decrease in arterial CO_2_ pressure (PaCO_2_) due to hyperventilation may be observed. At high exercise intensity, V'_E_ increases disproportionately to V'CO_2_, due to high levels of metabolic acidosis leading to a decrease in PaCO_2_ (Ramos et al., 2013; Sun et al., 2002). Individuals who chronically hyperventilate or have an elevated V_D_ typically exhibit higher V'_E_/V'CO_2_ values (Di Paolo et al., 2019; Neder et al., 2017; Ramos et al., 2013). In healthy sedentary individuals, the expected normal value for the V'_E_/V'CO_2_ slope, from unloaded pedaling to the ventilatory compensation point (VCP), is approximately 25, with 30 representing the upper limit of normal (Arena et al., 2008; Neder et al., 2001; Sun et al., 2002).

Conversely, lower V'_E_/V'CO_2_ slope values indicate a more efficient elimination of CO_2_ during exercise, suggesting a potential advantage to sports performance (Salazar‐Martínez et al., 2018). It is object of debate whether V'_E_/V'CO_2_ slope values may be predictive of exercise tolerance (Di Paco et al., 2017; Salazar‐Martínez et al., 2016) or fitness level (Salazar‐Martínez et al., 2018); while it does not seem to correlate with V'O_2_ max (Brown et al., 2012), BMI, or age (Salazar‐Martínez et al., 2018). Regarding the effect of sex differences, it has been shown that female athletes have higher slope values of V'_E_/V'CO_2_ compared to males (Collins et al., 2021; Guenette et al., 2007; Harms et al., 1998; Petek et al., 2023); these studies, however, have involved only a small number of young elite athletes, and they all focus primarily on endurance athletes and not on other sport categories.

The principal objective of our study was to prospectively analyze exercise ventilatory efficiency in a large cohort of elite athletes. We were specifically interested in establishing a frame of reference for this important variable, exposing the underlying factors that may modulate exertional ventilatory efficiency. We aimed to answer the following original research questions: (a) are there differences in ventilatory efficiency between male and female elite athletes? (b) is ventilatory efficiency modulated by ESC category? (Pelliccia et al., 2021); (c) can the different metrics of ventilatory efficiency (V'_E_/V'CO_2_ slope and the nadir value of V'_E_/V'CO_2_ ratio) be used interchangeably in elite athletes? and (d) are there physiological differences between subgroups of athletes stratified by ventilatory efficiency?

## 2 MATERIALS AND METHODS

### 2.1 Participants

Our study included a cohort of 443 Italian elite athletes (229 males and 214 females), selected for the 2023 European Games in Cracow (Poland) and potential candidates for the Paris 2024 Olympic Games. The study was conducted at the Institute of Sports Medicine and Science‐CONI, in Rome, Italy, between January and June 2023. This study received approval from the ethics committee (“Comitato Etico Territoriale Lazio Area 1”, approval number 0851/2024). Written informed consent was obtained from each participant. Patients or the public were not involved in the design, conduct or evaluation of the study.

### 2.2 Procedure

The athletes were stratified based on their predominant component of the ESC categories, as described by the 2020 ESC guidelines for exercise prescription (Pelliccia et al., 2021): (A) skill‐based (e.g., golf, sailing), (B) power‐based (e.g., weightlifting, boxing), (C) mixed (e.g., basketball, rugby, fencing), and (D) endurance‐focused activities (e.g., long distance running, cycling, rowing, triathlons). Pulmonary function tests, including spirometry and maximal voluntary ventilation (MVV) were obtained in each athlete. These measurements were obtained by using a pulmonary function testing system (Quark PFT, Cosmed, Pavona, Italy). The procedures followed the standards recommended by the American Thoracic Society (ATS) and the European Respiratory Society (ERS) (Graham et al., 2019). All measured variables were presented as absolute and percentage of the predicted values (adjusted for age, sex, BMI, and ethnicity) (Cooper et al., 2017). Each participant underwent a maximal incremental exercise test on an electronically braked cycle ergometer (Cosmed bike, Ergoselect 100, Ergoline, Bitz, Germany). The exercise protocol adhered to international recommendations (Radke et al., 2019; Weisman et al., 2001) and incorporated predicted normal values as outlined by Wasserman et al. (Radke et al., 2019; Sietsema et al., 2020; Weisman et al., 2001). The exercise phase involved incremental increases in work rate, ranging from 15 to 40 watts/min. These increments were tailored to the athletes' physical performance and the predicted V'O_2_ peak, aiming to achieve maximal exertion within the 8‐ to 12‐min timeframe. Electrocardiogram (ECG) data were continuously monitored throughout both maximal exercise and the subsequent 5‐min recovery period. A metabolic cart was utilized for gas exchange analysis (Quark CPET, Cosmed, Pavona, Italy). The following variables were measured breath‐by‐breath and then averaged every 20 s: V'O_2_, V'CO_2_, tidal volume, breathing frequency, V'_E_, end tidal O_2_ (PetO_2_), and end tidal CO_2_ (PetCO_2_). The oxygen pulse was calculated using the ratio V'O_2_ over heart rate HR (V'O_2_/HR). The lactate threshold (LT) was identified using both the V‐slope and ventilatory equivalents methods (Sietsema et al., 2020). The VCP was determined by analysing the relationship between V'_E_ and V'CO_2_ over time, following established standard criteria (Sietsema et al., 2020). Ventilatory efficiency was assessed by using: (a) the V'_E_/V'CO_2_ slope (V'_E_/V'CO_2_ s_1_) and the intercept (V'_E_/V'CO_2_ I), with the exclusion of the data above the VCP; (b) V'_E_/V'CO_2_ value at the LT (V'_E_/V'CO_2_@LT); (c) the V'_E_/V'CO_2_ value at the nadir (V'_E_/V'CO_2_ nadir); (d) the V'_E_/V'CO_2_ slope above the VCP (V'_E_/V'CO_2_ s_2_).

### 2.3 Statistical analysis

Descriptive statistics, such as means and standard deviations, were computed for essential CPET parameters to provide a summary of central tendency and variability within the dataset. To assess differences in CPET results between males and females, as well as across sport categories, the unpaired Student's *t*‐test was employed. Additionally, group comparisons were conducted using ANOVA. Linear regression and the Bland–Altman plot were employed to assess the agreement between V'_E_/V'CO_2_ s_1_ and nadir in evaluating ventilatory efficiency. A significance level of *p* < 0.05 was set as the threshold for determining statistical significance. All statistical analyses were carried out using Microsoft Excel and GraphPad Prism.

## 3 RESULTS

Baseline and spirometric characteristics of all the participants are shown in Table 1. The average age of the population was 25 ± 5 years, with a similar distribution between males and females. In contrast, males exhibited higher values of height and BMI compared to females. As expected, spirometric absolute values forced vital capacity (FVC), forced expiratory volume in the first second (FEV_1_), forced mid‐expiratory flow (FEF_25‐75_) and MVV were higher in males than in females, while the percentage of predicted values was similar and higher than normal for both genders (Graham et al., 2019).

**TABLE 1:** Baseline and spirometric characteristics.

	Total	Males	Females
Number of subjects	443	229	214
Age, years	25 ± 5	26 ± 6	25 ± 5
Height, cm	175 ± 10	182 ± 9	169 ± 7
BMI, kg/m^2^	23.4 ± 2.9	24.3 ± 3.0	22.5 ± 2.6
ESC classification			
A	115 (26)	55 (24)	60 (28)
B	76 (17)	40 (17)	36 (17)
C	190 (43)	100 (44)	190 (42)
D	62 (14)	34 (15)	28 (13)
Hb, mg/dL	14.1 ± 1.4	14.9 ± 1.3	13.3 ± 1.0
FVC, l (%)	5.1 ± 1.1 (103 ± 14)	5.8 ± 0.9 (102 ± 11)	4.4 ± 0.7 (105 ± 16)
FEV_1_, l (%)	4.2 ± 0.8 (102 ± 12)	4.8 ± 0.7 (101 ± 11)	3.7 ± 0.5 (103 ± 13)
FEV_1_/FVC, %	83 ± 7	82 ± 7	84 ± 6
FEF_25‐75_, l (%)	4.4 ± 1.2 (99 ± 22)	4.9 ± 1.3 (99 ± 25)	3.9 ± 0.8 (98 ± 20)
MVV, l	145.6 ± 33.0	169.3 ± 26.7	122.9 ± 19.9

*Note*: Data are presented as mean ± standard deviation.

Abbreviations: BMI, Body Mass Index; FEF_25‐75_, Forced Expiratory Flow at 25%–75%; FEV_1_, Forced Expiratory Volume in the first second; FVC, Forced Vital Capacity; Hb, Hemoglobin; MVV, Maximum Voluntary Ventilation.

### 3.1 Physiological variables at CPET

Compared to males, females exhibited lower exercise tolerance, as evidenced by significantly lower values in V'O_2_ peak (*p* < 0.001), V'O_2_/kg peak (*p* < 0.001), and maximal power (*p* < 0.001) (Table 2). Conversely, the ratio of increase of V'O_2_ work rate relationship (V'O_2_/WR) appeared to be independent of sex or ESC category. Furthermore, at peak exercise, males showed lower HR values, higher V'O_2_/HR, and higher V'_E_. As shown in Table 3, endurance athletes (ESC D) had a significantly higher V'O_2_ peak, V'O_2_/kg peak and maximal power, compared to other ESC categories. In addition, ESC D athletes had a better cardiovascular performance (i.e., V'O_2_/HR and HR peak), and higher V'_E_ peak; this was particularly evident when compared to skill athletes (ESC A).

**TABLE 2:** Physiologic variables at CPET.

	Total (443)	Males (229)	Females (215)	*p* Value
Maximal power, watt	242 ± 68	283 ± 61	199 ± 45	<0.001
V'O_2_ peak, mL/min	2970 ± 743	3438 ± 630	2472 ± 486	<0.001
V'O_2_/Kg peak, mL/min/kg	41.4 ± 9.1	43.6 ± 10.0	39.1 ± 7.4	<0.001
V'O_2_ peak, % predicted	118.4 ± 23.5	108.3 ± 18.4	129.2 ± 23.6	<0.001
V'CO_2_ peak, mL/min	3282 ± 830	3836 ± 659	2689 ± 532	<0.001
RER	1.12 ± 0.08	1.14 ± 0.08	1.11 ± 0.07	<0.001
V'O_2_/WR, mL/watt	10.1 ± 0.9	10.1 ± 0.8	10.1 ± 0.9	NS
HR peak, bpm	167 ± 11	165 ± 11	169 ± 10	<0.001
V'O_2_/HR, mL/bpm	17.9 ± 4.7	20.9 ± 4.1	14.7 ± 3.0	<0.001
V'_E_ peak, L/min	100.5 ± 26.0	116.2 ± 24.1	83.9 ± 16.4	<0.001
BR, L/min	30.6 ± 25.0	30.8 ± 30.4	30.4 ± 17.7	NS
V'_E_/V'CO_2_ s_1_	25.5 ± 2.9	24.7 ± 2.6	26.3 ± 2.9	<0.001
V'_E_/V'CO_2_@LT	25.8 ± 2.7	25.0 ± 2.6	26.7 ± 2.5	<0.001
V'_E_/V'CO_2_ nadir	25.0 ± 2.4	24.3 ± 2.1	25.8 ± 2.4	<0.001
V'_E_/V'CO_2_ i	3.2 ± 2.1	3.4 ± 2.2	2.9 ± 2.0	<0.05
V'_E_/V'CO_2_ s_2_	45.8 ± 19.5	46.8 ± 25.2	44.6 ± 8.7	NS
Power @LT, watt	125 ± 50	146 ± 52	103 ± 38	<0.001
VO_2_@LT, mL/min	1775 ± 555	2011 ± 550	2967 ± 653	<0.001
Power @RCP, watt	206 ± 64	241 ± 59	165 ± 42	<0.001
VO_2_@VCP, mL/min	2583 ± 710	1525 ± 439	2132 ± 469	<0.001

*Note*: Data are presented as mean ± standard deviation.

Abbreviations: BR, Breathing Reserve; i, intercept; LT, lactate threshold; RER, Respiratory Exchange Ratio; V'CO_2_, carbon dioxide output; V'O_2_, oxygen uptake; VCP, Ventilatory Compensation Point; V'_E_, minute ventilation; WR, Work Rate.

**TABLE 3:** Physiologic variables at CPET—sport categories.

		Total	ESC A (115)	ESC B (76)	ESC C (190)	ESC D (62)	*p* Value
Sex							
Males (%)		229 (52)	55 (48)	40 (53)	100 (53)	34 (55)	
Females (%)		214 (48)	60 (52)	36 (47)	90 (47)	28 (45)	
Maximal power, watt		242 ± 68	195 ± 49	238 ± 59*	255 ± 57*†	297 ± 83*^†‡^	<0.001
V'O_2_ peak, mL/min		2970 ± 743	2445 ± 570	2986 ± 676*	3112 ± 637*	3496 ± 843*^†‡^	<0.001
V'O_2_/Kg peak, mL/min/kg		41.4 ± 9.1	36.4 ± 7.2	42.0 ± 6.5*	40.8 ± 5.4*	51.0 ± 11.8*^†‡^	<0.001
V'O_2_ peak, % predicted		118.4 ± 23.5	103.3 ± 17.9	119.0 ± 17.8*	120.3 ± 20.0*	139.9 ± 29.7*^†‡^	<0.001
V'CO_2_ peak, mL/min		3282 ± 830	2763 ± 636	3332 ± 782*	3402 ± 761*	3830 ± 921*^†‡^	<0.001
RER		1.12 ± 0.08	1.15 ± 0.09	1.13 ± 0.08	1.11 ± 0.07*	1.11 ± 0.08*	NS
V'O_2_/WR, mL/watt		10.1 ± 0.9	10.1 ± 1.0	10.4 ± 0.9	10.0 ± 0.8^†^	10.0 ± 0.7^†^	<0.05
HR peak, bpm		167 ± 11	170 ± 11	163 ± 11*	166 ± 10*	166 ± 11*	<0.001
V'O_2_/HR, mL/bpm		17.9 ± 4.7	14.4 ± 3.4	18.5 ± 5.2*	18.8 ± 4.0*	20.8 ± 4.9*^†‡^	<0.001
V'_E_ peak, L/min		100.5 ± 26.0	87.0 ± 19.3	101.6 ± 29.5*	104.6 ± 24.9*	112.2 ± 28.0*^†‡^	<0.001
BR, L/min		30.6 ± 25.0	36.9 ± 13.9	28.8 ± 26.5*	30.7 ± 16.6*	21.0 ± 48.0*	<0.001
V'_E_/V'CO_2_ s_1_		25.5 ± 2.9	25.9 ± 3.1	25.2 ± 3.0	25.5 ± 2.7	25.0 ± 2.5*	NS
V'_E_/V'CO_2_ LT		25.8 ± 2.7	26.4 ± 2.8	25.3 ± 2.3*	25.9 ± 2.9	25.1 ± 2.1*^‡^	<0.05
V'_E_/V'CO_2_ nadir		25.0 ± 2.4	25.4 ± 2.6	24.7 ± 2.3	25.1 ± 2.3	24.5 ± 2.1*	NS
V'_E_/V'CO_2_ interc.		3.2 ± 2.1	2.8 ± 2.0	3.0 ± 2.1	3.4 ± 2.3*	3.1 ± 1.8	NS
V'_E_/V'CO_2_ s_2_		45.8 ± 19.5	44.6 ± 8.5	50.8 ± 42.8*	45.1 ± 9.4	44.4 ± 8.4	NS
Power @LT, watt		125 ± 50	92 ± 27	121 ± 36*	131 ± 40*	171 ± 79*^†‡^	<0.001
V'O_2_ @LT, mL/min		1775 ± 555	1428 ± 321	1750 ± 429*	1846 ± 489*	2239 ± 784*^†‡^	<0.001
Power @RCP, watt		206 ± 64	161 ± 46	206 ± 53*	218 ± 55*	257 ± 80*^†‡^	<0.001
V'O_2_ @RCP, mL/min		2583 ± 710	2094 ± 545	2620 ± 637*	2715 ± 608*	3078 ± 837*^†‡^	<0.001

*p < 0.05 versus ESC A, using post hoc test. ^†^ *p* < 0.05 versus ESC B, using post hoc test. ^‡^ *p* < 0.05 versus ESC C, using post hoc test.

*Note*: Data are presented as mean ± standard deviation.

Abbreviations: BR, Breathing Reserve; I, intercept; LT, lactate threshold; RER, Respiratory Exchange Ratio; V'CO_2_, carbon dioxide output; V'O_2_, oxygen uptake; VCP, Ventilatory Compensation Point; V'_E_, minute ventilation; WR, Work Rate.

### 3.2 Ventilatory efficiency

**FIGURE 1:**
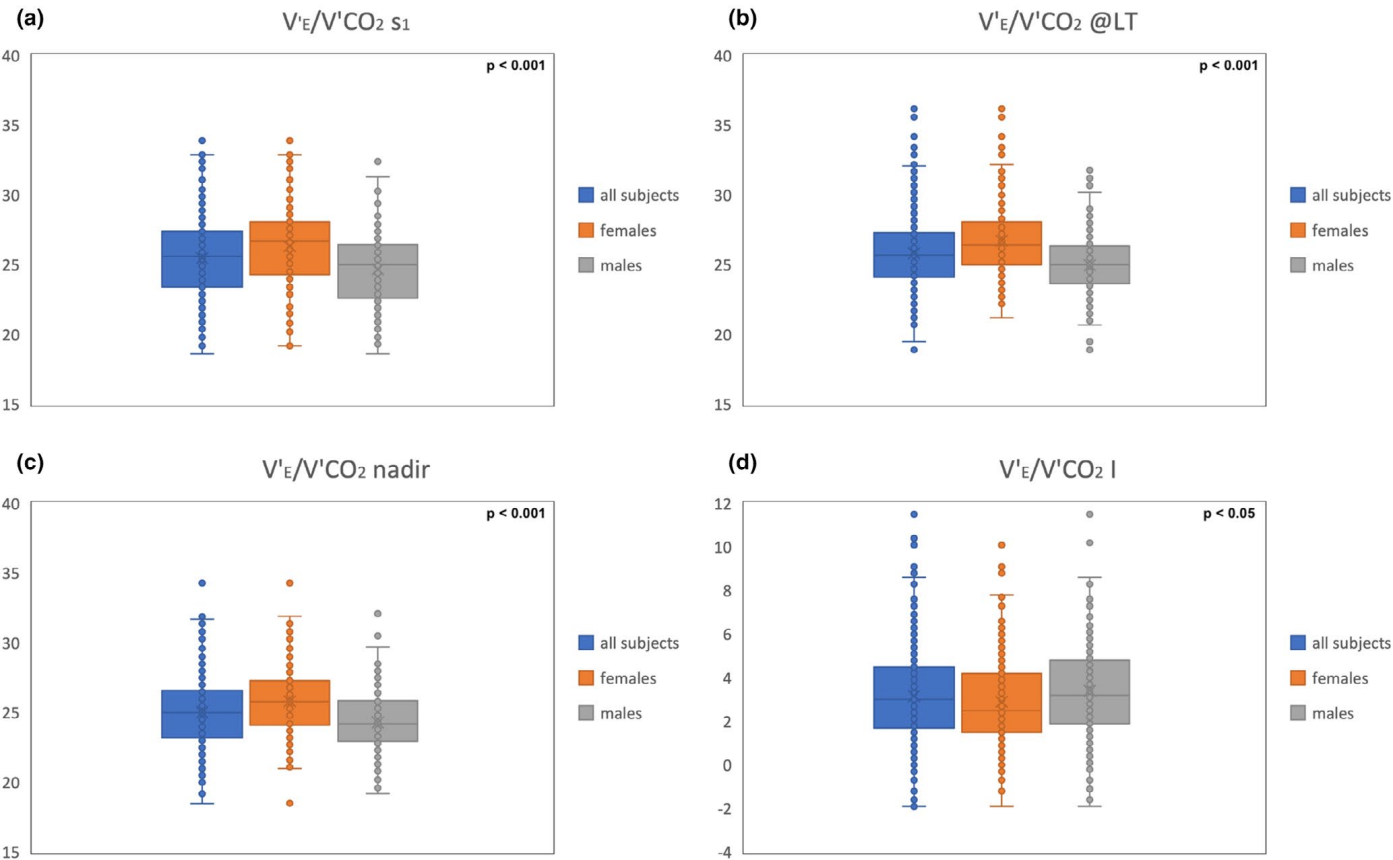
Comparison of V'_E_/V'CO_2_ slope (a), at the lactate threshold (b), nadir (c) and intercept (d) between females and males. The *y*‐axis reports the values of V'_E_/V'CO_2_. V'CO_2_, carbon dioxide output; V'_E_, minute ventilation; S_1_, slope before the ventilatory compensation point; LT, lactate threshold; I, intercept.

As shown in Table 2 and Figure 1, female athletes showed lower ventilatory efficiency, with higher values of V'_E_/V'CO_2_ s_1_ (*p* < 0.001), V'_E_/V'CO_2_ nadir (*p* < 0.001), V'_E_/V'CO_2_@AT (*p* < 0.001), and V'_E_/V'CO_2_ i (*p* < 0.05). Both males and females of the subgroup of endurance athletes (ESC D) exhibited higher ventilatory efficiency, with lower values for V'_E_/V'CO_2_ s_1_ (*p* < 0.001), V'_E_/V'CO_2_ nadir (*p* < 0.001), V'_E_/V'CO_2_@LT (*p* < 0.001), and V'_E_/V'CO_2_ i (*p* < 0.001), compared to skill athletes (ESC A) (Table 3).

**FIGURE 2:**
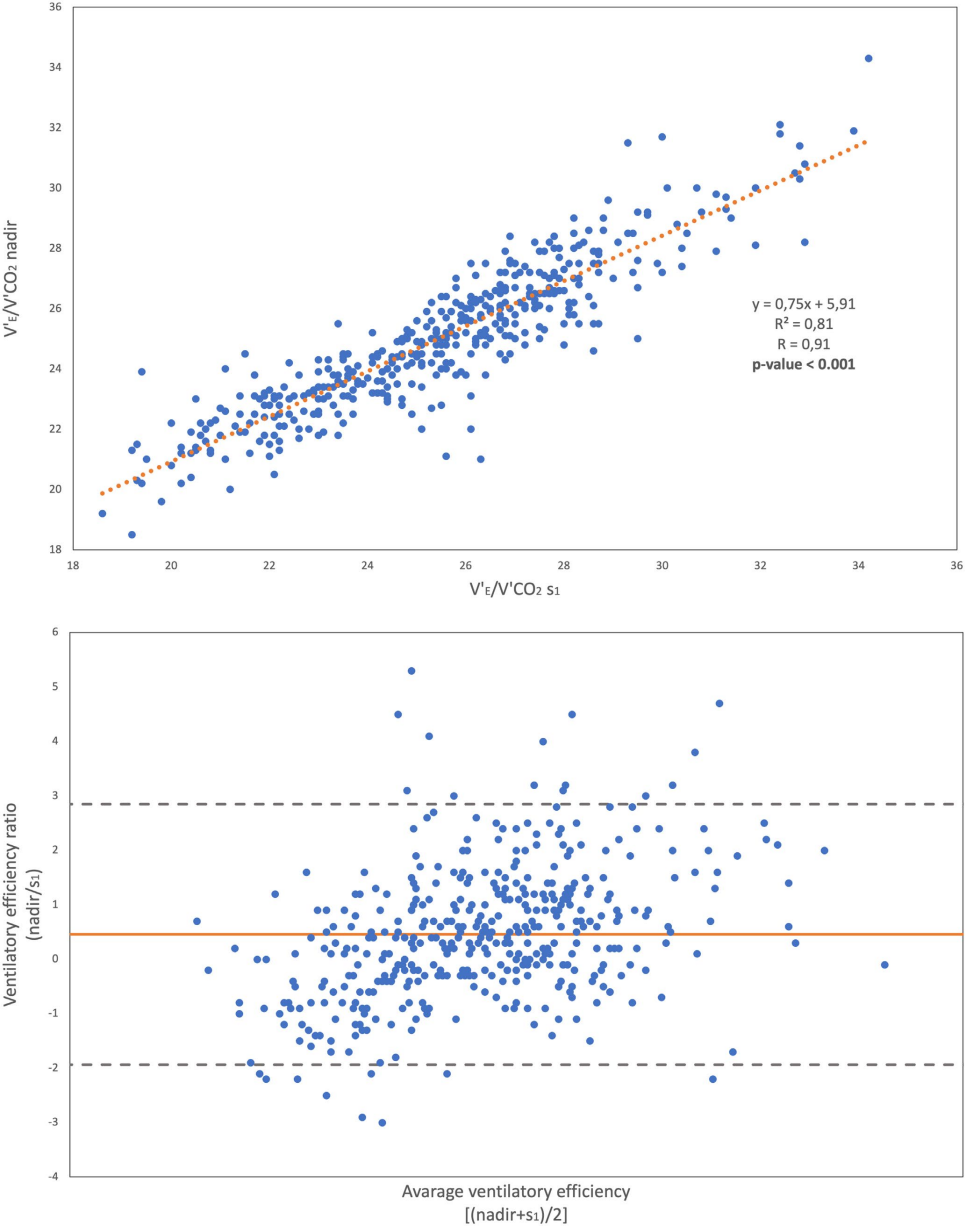
Upper panel: Linear regression, comparison between V'_E_/V'CO_2_ nadir versus V'_E_/V'CO_2_ s_1_ values. Lower panel: Bland–Altman plot, comparison between V'_E_/V'CO_2_ nadir versus V'_E_/V'CO_2_ s_1_ values; V'CO_2_, carbon dioxide output; V'_E_, minute ventilation; S_1_, slope before the ventilatory compensation point.

A strong positive correlation (Figure 2a) was observed between V'_E_/V'CO_2_ s_1_ and V'_E_/V'CO_2_ nadir for all athletes (*r* = 0.91; *p* < 0.001). By using the Bland–Altman test, we found a good agreement between V'_E_/V'CO_2_ s_1_ and V'_E_/V'CO_2_ nadir in determining ventilatory efficiency (Figure 2b). A minimal deviation from the line of equality was observed and, on average, the difference between the nadir values minus the s_1_ values was only 0.46. The mean bias of the two measurements, defined as ±1.96 standard deviations, were −1.94 and 2.85. A total of 420 measurements out of 443 (99.5%) fell within this interval.

### 3.3 Ventilatory efficiency below and above limits of normality

Two subgroups of athletes were identified based on their V'_E_/V'CO_2_ s_1_ values: one with high values and another with low values. The cutoff values were chosen in accordance with the upper limit of normality (V'_E_/V'CO_2_ s_1_ > 32) (Radke et al., 2019; Sietsema et al., 2020; Weisman et al., 2001) and −1 standard deviation from the mean value of the entire population (V'_E_/V'CO_2_ s_1_ < 22).

Fifty‐one athletes (11.7%) had a V'_E_/V'CO_2_ s_1_ lower than 22 while eight athletes (1.8%) had a V'_E_/V'CO_2_ s_1_ higher than 32 (Table 4). In the two subgroups, V'_E_/V'CO_2_ s_2_ (*p* < 0.05), V'_E_/V'CO_2_ nadir (*p* < 0.001), V'_E_/V'CO_2_@LT (*p* < 0.001), and V'_E_/V'CO_2_ i (*p* < 0.001) were also statistically different. Furthermore, in athletes with V'_E_/V'CO_2_ s_1_ < 22, PetCO_2_ was significantly higher at rest (*p* < 0.001), LT (*p* < 0.001), nadir (*p* < 0.001), and at peak work rate (*p* < 0.001) compared to those athletes with V'_E_/V'CO_2_ s_1_ > 32. Of note, the group of athletes exhibiting V'_E_/V'CO_2_ s_1_ < 22 showed significantly higher V'O_2_ peak, V'O_2_/kg peak, and maximal power compared to the group with V'_E_/V'CO_2_ s_1_ higher than 32. Watt max of the high ventilatory efficiency group was also significantly higher compared to all athletes studied (see Table 2; *p* < 0.05).

**TABLE 4:** Ventilatory efficiency below and over limits of normality.

	VE/VCO2 < 22	VE/VCO2 > 32	*p* Value
Number of subjects (%)	51 (11.7)	8 (1.8)	
Age, years	24.8 ± 5.5	25.0 ± 7.2	
Sex			
Males (%)	14 (26.9)	1 (12.5)	
Females (%)	37 (71.1)	7 (87.5)	
Height, cm	178 ± 10	173 ± 6	
Weight, kg	70.8 ± 9.3	72.9 ± 13.9	
Comorbidities			
Asthma	1 (2%)	1 (13%)	NS
Smoking history	0 (0%)	2 (33%)	NS
Hypertension	0 (0%)	0 (0%)	NS
Diabetes	0 (0%)	1 (13%)	NS
Dyslipidemia	1 (2%)	0 (0%)	NS
Estroprogestinic therapy	6 (12%)	3 (38%)	**<0.05**
Maximal power, watt	260 ± 60	205 ± 48	**<0.05**
V'O_2_ peak, mL/min	3064 ± 723	2526 ± 423	**<0.05**
V'O_2_/Kg peak, mL/min/kg	42.8 ± 8.7	35.8 ± 5.0	**<0.05**
V'O_2_ peak, % predicted	111.5 ± 8.7	122.5 ± 24.0	NS
V'_E_/V'CO_2_ s_1_	20.7 ± 0.9	33.0 ± 0.7	**<0.001**
Intercept	5.9 ± 1.9	1.0 ± 1.6	**<0.001**
V'_E_/V'CO_2_ LT	23.3 ± 1.8	32.0 ± 2.3	**<0.001**
V'_E_/V'CO_2_ nadir	21.8 ± 1.2	31.0 ± 1.7	**<0.001**
V'_E_/V'CO_2_ s_2_	42.0 ± 10.6	53.0 ± 7.0	**<0.05**
PetCO_2_ rest, mmHg	34.3 ± 2.7	30.0 ± 4.3	**<0.001**
PetCO_2_ LT, mmHg	46.8 ± 3.3	35.0 ± 2.6	**<0.001**
PetCO_2_ nadir, mmHg	50.5 ± 3.0	35.0 ± 2.2	**<0.001**
PetCO_2_ peak of exercise, mmHg	44.3 ± 4.3	32.0 ± 2.2	**<0.001**
Delta PetCO_2_, mmHg	16.2 ± 3.8	2.0 ± 5.0	**<0.001**

*Note*: Data are represented as mean ± standard deviation.

Abbreviations: Delta, PetCO_2_@nadir – PetCO_2_@rest; I, intercept; LT, lactate threshold; V'CO_2_, carbon dioxide output; V'O_2_, oxygen uptake; V'_E_, minute ventilation.

### 3.4 Ventilatory efficiency as a continuum

To further investigate the potential link between ventilatory efficiency and exercise tolerance, the athletes were also divided into tertiles based on their V'_E_/V'CO_2_ s_1_. The three subgroups were defined as follows: V'_E_/V'CO_2_ s_1_ < 24, between 24 and 27, and >27. A total of 132 athletes (29.8%) were classified in the first group, 186 athletes (42.0%) in the second group, and 125 athletes in the third group (28.2%). Importantly, progressively higher ventilatory efficiency was associated with higher V'O_2_ peak (*p* < 0.001), V'O_2_/kg peak (*p* < 0.001), maximal power (*p* < 0.001), and V'O_2_/HR (*p* < 0.05) (Table 5).

**TABLE 5:** Ventilatory efficiency as a continuum.

	V'_E_/V'CO_2_ < 24	V'_E_/V'CO_2_ 24–27	V'_E_/V'CO_2_ > 27	*p* Value
Number of subjects (%)	132 (29.8)	186 (42.0)	125 (28.2)	
Sex				
Males (%)	84 (64)	105 (56)	40 (32)	
Female (%)	48 (36)	81 (44)	85 (68)	
ESC classification (%)				
A	33 (23)	46 (25)	38 (30)	
B	27 (20)	27 (15)	22 (18)	
C	57 (43)	80 (43)	53 (42)	
D	17 (13)	33 (18)	12 (10)	
Age, years	25 ± 5	25 ± 5	25 ± 6	NS
Height, cm	177 ± 10	176 ± 10	173 ± 10	**<0.05**
BMI, kg/m^2^	23.3 ± 2.5	23.6 ± 2.8	23.4 ± 3.7	NS
Maximal power, watt	257 ± 62	246 ± 71	221 ± 64*^†^	**<0.001**
V'O_2_ peak, mL/min	3090 ± 696	3038 ± 781	2746 ± 686*^†^	**<0.001**
V'O_2_/Kg peak, mL/min/kg	42.7 ± 8.6	41.6 ± 8.5	39.4 ± 7.9*	**<0.001**
V'O_2_ peak, % predicted	117.4 ± 25.6	117.6 ± 22.3	120.6 ± 22.9	NS
V'CO_2_ peak, mL/min	3505 ± 762	3358 ± 834	2959 ± 747*	**<0.001**
RER	1.15 ± 0.08	1.12 ± 0.07*	1.09 ± 0.07*^†^	**<0.05**
V'O_2_/WR, mL/watt	10.1 ± 0.9	10.1 ± 0.8	10.2 ± 0.9	NS
HR peak, bpm	167 ± 10	167 ± 11	166 ± 12	NS
V'O_2_/HR, mL/bpm	18.5 ± 4.3	18.2 ± 4.9	16.7 ± 4.5*^†^	**<0.05**
VE peak, L/min	97.9 ± 24.2	102.6 ± 26.0	100.3 ± 28.6	NS
BR, L/min	33.5 ± 34.7	30.5 ± 19.9	27.0 ± 17.8	NS
V'_E_/V'CO_2_ s_2_	44.9 ± 32.7	44.3 ± 8.2	49.3 ± 8.8	NS

*p < 0.05 versus V'_E_/V'CO_2_ < 24, using post hoc test. ^†^ *p* < 0.05 versus V'_E_/V'CO_2_ 24–28, using post hoc test.

*Note*: Data are presented as mean ± standard deviation.

Abbreviations: BR, Breathing Reserve; I, intercept; LT, lactate threshold; RER, Respiratory Exchange Ratio; V'CO_2_, carbon dioxide output; V'O_2_, oxygen uptake; VCP, Ventilatory Compensation Point; V'_E_, minute ventilation; WR, Work Rate.

## 4 DISCUSSION

This is the largest study to assess ventilatory efficiency as related to exercise performance in elite athletes evaluated by CPET. It provides a comprehensive analysis of maximal exercise tolerance and, more importantly, the ventilatory and pulmonary gas exchange adaptations to maximal incremental exercise. Notably, ventilatory efficiency emerged as a potentially critical correlate of athletic performance, highlighting its practical importance beyond conventional cardiopulmonary fitness parameters.

### 4.1 Sex differences

One of the main results of our study was the observed difference in ventilatory efficiency between male and female athletes. Females exhibited higher exercise V'_E_/V'CO_2_ values, indicating less efficient CO_2_ elimination compared to males. These results are in accordance with previous data from literature in non‐athletic populations (Collins et al., 2021; Neder et al., 2001; Sietsema et al., 2020). Several studies have suggested that this difference arises from the mechanical disadvantage that females face during exercise, due to narrower airways and smaller lung volumes (Collins et al., 2021; Dominelli et al., 2013; Guenette et al., 2009; Kilbride et al., 2003; Neder et al., 2001; Sietsema et al., 2020). These physiological factors lead to a higher resistive work of breathing and an increased dead space ventilation tidal volume ratio (V_d_/V_t_) during exercise; females tend to adopt a more tachypneic breathing pattern, characterized by lower tidal volumes and higher breathing frequency (Collins et al., 2021; Dominelli et al., 2013; Guenette et al., 2009; Kilbride et al., 2003; Neder et al., 2001; Sietsema et al., 2020). Additionally, a lower CO_2_ set point in females compared to males has been proposed as another potential factor contributing to the reduced ventilatory efficiency (Collins et al., 2021; Dominelli et al., 2013; Kilbride et al., 2003; Neder et al., 2001; Sietsema et al., 2020).

### 4.2 Athletes stratified by ESC categories

Importantly, we also observed differences in ventilatory efficiency among different ESC sport categories. Notably, endurance athletes (ESC D) exhibited higher ventilatory efficiency compared to athletes engaged in skill‐based sports (ESC A). It is possible to hypothesize that a more efficient pulmonary gas exchange during exercise is needed to accomplish the high aerobic demands of endurance sport activities. Future studies, however, are needed to understand whether high ventilatory efficiency in these athletes is genetically based (e.g., neural control of breathing) and/or could be influenced by specific training programs.

We also found that peak V'O_2_, V'O_2_/kg, and V'O_2_/HR (O_2_ pulse) values are markedly increased in the ESC D category compared to the other categories. Research data on this topic are limited. Our findings, however, are in agreement with previous literature, which shows the enhanced cardiovascular and oxygen‐carrying ability and/or oxygen extraction capacity observed in athletes who perform aerobic training (Adami et al., 2022; Degens et al., 2019).

Moreover, in our study, the V'O_2_/WR relationship was found to be independent of both sex and ESC categories. This confirms data from the literature describing mechanical efficiency (V'O_2_/WR relationship) to be independent of age, sex, and height (Barron et al., 2015; Sietsema et al., 2020), while the role of fitness level or training in changing this relationship is still debated. Some studies showed higher slopes in trained cyclists (Sietsema et al., 2020) and lower ones in less fit subjects (Neder et al., 2001). On the other hand, it has been stated that lower V'O_2_/WR values could represent a potential advantage for endurance athletes, resulting in a lower percentage of V'O_2_ max utilization per work rate (Mazaheri et al., 2021).

### 4.3 Athletes stratified by ventilatory efficiency

Another important finding of our study is that we were able to identify a significant subset of athletes, 51 out of 444 athletes (11.7%), showing particularly high ventilatory efficiency (i.e., V'_E_/V'CO_2_ s1 < 22), which is indicative of a different strategy in eliminating CO_2_ during exercise. This level of efficiency seemed to be advantageous, as it was associated with greater exercise performance; in fact, when all athletes were divided into three tertiles, according to the levels of ventilatory efficiency, this finding was also confirmed. However, the direct correlation between high ventilatory efficiency and sports performance outcomes remains underexplored and forms a critical avenue for future research.

Our results are in agreement with the work of Bussotti and colleagues that described (Bussotti et al., 2008), in healthy young individuals, a higher exercise tolerance and maximal aerobic capacity in those subjects with a blunted ventilatory response to exercise associated with higher end‐tidal CO_2_. As shown in Table 3, athletes of ESC D category had higher V'O_2_ peak associated with higher ventilatory efficiency, that is, lower V'_E_/V'CO_2_@LT. In addition, as shown in Table 3, subjects with very high ventilatory efficiency (V'_E_/V'CO_2_ s_1_ < 22) had a higher V'O_2_ peak compared with those with low ventilatory efficiency (V'_E_/V'CO_2_ s_1_ > 32). These findings support the hypothesis, originally proposed by Harms and colleagues (Harms et al., 1997; Harms & Stager, 2005), that a blunted ventilatory response to exercise, with associated lower respiratory muscle work, has a favorable effect on muscle leg blood flow of exercising muscles.

### 4.4 Clinical implications

Looking forward, the potential implications of these findings are vast. Establishing a link between ventilatory efficiency and performance could change training strategies and athlete assessment protocols. Moreover, comparing these CPET‐derived parameters across various disciplines could provide deeper insights into how athletes can optimize performance through targeted interventions (e.g., training, diet, etc). Such studies could also stress the use of CPET as a routine part of athletic physical performance evaluation and monitoring, not just for clinical assessments.

## 5 CONCLUSIONS

In conclusion, our study not only contributes to the current understanding of cardiopulmonary responses in elite athletes but also opens new insights for the analysis of ventilatory efficiency in sports medicine.

## AUTHOR CONTRIBUTIONS

Paolo Palange and Maria Rosaria Squeo had the idea and planned the study. Ilaria Menichini, Matteo Morviducci, Alessandro Spinelli, Giuseppe Di Gioia, and Viviana Maestrini performed the tests. Matteo Morviducci, Ilaria Menichini, and Alessandro Spinelli collected the data. Paolo Palange, J Alberto Neder, Ilaria Menichini, and Matteo Morviducci analysed the data. Paolo Palange, Matteo Morviducci, and Ilaria Menichini made the Tables and Figures. Paolo Palange, Maria Rosaria Squeo, J Alberto Neder, and Matteo Morviducci contributed to the writing of the manuscript.

## FUNDING INFORMATION

No funding was received.

## CONFLICT OF INTEREST STATEMENT

We confirm that none of the authors has any conflict of interest.

## ETHICS STATEMENT

This study received approval from the ethics committee (“Comitato Etico Territoriale Lazio Area 1”, approval number 0851/2024). Written informed consent was obtained from each participant.

## Data Availability

The data that support the findings of this study are available on request from the corresponding author. The data are not publicly available due to privacy.
